# Response to the Comment on “Bariatric Surgery can Lead to Net Cost Savings to Health Care Systems: Results from a Comprehensive European Decision Analytic Model”

**DOI:** 10.1007/s11695-015-1695-6

**Published:** 2015-05-01

**Authors:** Oleg Borisenko, Daniel Adam, Peter Funch-Jensen, Ahmed R. Ahmed, Rongrong Zhang, Zeynep Colpan, Jan Hedenbro

**Affiliations:** Synergus AB, Svardvagen 19, 182 33 Danderyd, Sweden; Aarhus University, Aarhus, Denmark; Aleris Hamlet Hospital Aarhus, Aarhus, Denmark; Imperial College London, London, UK; Aleris Obesity, Lund, Sweden; Lund University, Lund, Sweden

Dear Editor,

We are grateful for having the possibility to comment on the “Letter to the editor” regarding our article “Bariatric Surgery can Lead to Net Cost Savings to Health Care Systems: Results from a Comprehensive European Decision Analytic Model”.

Fildes et al. have compared our data and results with those from the Swedish Obese Subjects study [1]. Our results are to 98 % based on gastric bypass surgery, the dominating procedure in Sweden and in Europe. The SOS study comprises only 13 % gastric bypass patients, with the majority of patients operated with purely restrictive operations, 68 % vertical banded gastroplasty (VBG) and 19 % gastric banding. It has been clearly shown that VBG is accompanied by high costs, since up to 89 % of patients need a conversion [2]. In our analysis, reduction of use of gastric bypass with corresponding increase in other types of surgeries led to reduction of cost-saving effect.

The comparison with the report by Weiner et al. [3] is not quite appropriate either. That study has observed costs over a 6-year period for a group of bariatric surgery patients, most of whom had had a gastric bypass, laparoscopic (38.3 %) or open (35.4 %). However, the control group was chosen because patients had diseases that were obesity-related. The study reported a significant loss to follow-up over 6 years. Total costs were higher in years 2 and 3 for the surgery group, but these differences disappeared with time. Weiner et al. did not have data beyond year 6. Since we report that the economic benefit of surgery to health care system appears after 17 years, we believe the results from the Weiner’s study do not contradict our conclusion.

Limited duration of follow-up is an obvious and most common limitation of published empirical research of economic consequences of bariatric surgery. Due to the initial high cost of surgery, cumulative cost of surgical treatment has shown to be higher compared with cumulative cost of alternative conservative management in a short- and mid-term time horizon. To overcome this limitation and to incorporate long-term consequence of treatments, we have employed lifetime horizon in our model.

Fildes et al. had commented on omitting some of obesity-related costs in our analysis, which can contribute to cost-saving effect. We have considered the costs, related to surgery, surgical complications, treatment of diabetes and cardiovascular disorders. These costs constitute about 80 % of total obesity-related cost as demonstrated in earlier [4–6] studies, although was reported to be lower in more recent studies [7]. Swedish cost-of-illness studies in obesity have also focused only on diabetes and cardiovascular outcomes [8]. In our opinion, the most important costs are included into analysis. Nevertheless, we agree that that with evolvement of treatments (e.g. for cancer), the relative weight of these cost items may be greater over the time.

We agree with the comment that validation of the model with studies of bariatric surgery is of high value. In our analysis, incidence and remission of diabetes were validated with the Scandinavian Obesity Surgery Registry, and the number of cardiovascular outcomes and mortality was validated with the Action for Health in Diabetes (AHEAD) study of lifestyle interventions, which included 5145 overweight or obese patients with type 2 diabetes (start BMI for validation was 35.9 kg/m^2^) [9]. It is acknowledged that there would always be not enough validation attempts, as clinical data evolve over time. We agree that additional specific validation exercises would be of benefit.

In their comment, Fildes et al. focused on the overall conclusion from the analysis, although important sub-group differences in economic outcomes were shown. Thus, cost-saving effect was reported in the base-case analysis (with characteristics of the cohort, mimicking characteristics of real surgical candidates in Sweden) and single-cohort analysis of diabetic patients and severe to super obese non-diabetic patients. But in single-cohort analysis, we did not observe cost savings in patients with moderate and severe obesity and no diabetes at baseline.

Bariatric surgery is a good example of a rapidly evolving area with changes of surgical techniques, dramatic reduction in the use of open surgical approach and improved safety. Empirical economic data, although being the source of information of the greatest validity, are always available with delay, usually have limited follow-up and often do not correspond to the current treatment practice. In this case, decision analytic modelling has an inevitable complementary role to inform clinical and policy decision-making, acknowledging its simplification over reality. Omitting data generated by comprehensive modelling would lead to underestimation of true economic value of modern bariatric surgery.
